# Sensitization to Hymenoptera venom in pollen allergic patients: Frequency and involvement of cross-reacting carbohydrate determinants (CCD)

**DOI:** 10.1371/journal.pone.0238740

**Published:** 2020-09-08

**Authors:** Katrin Bergmann-Hug, Michael Fricker, Oliver Hausmann, Arthur Helbling, Lukas Jörg

**Affiliations:** 1 Allergy Unit, Zieglerspital, Clinic of Internal Medicine, Spital Netz Bern AG, Bern, Switzerland; 2 Department of Rheumatology, Immunology and Allergology, Inselspital, Bern University Hospital, University of Bern, Bern, Switzerland; 3 Praxisgemeinschaft Mörigen, Mörigen, Switzerland; 4 ADR-AC GmbH, Adverse Drug Reactions, Analysis and Consulting, Bern, Switzerland; 5 Löwenpraxis Luzern, Lucerne, Switzerland; Copenhagen University Hospital Holbæk, DENMARK

## Abstract

Sensitization to Hymenoptera venom in patients without a history of systemic allergic reactions to Hymenoptera stings is frequently found and can be due to the presence of specific IgE to cross-reactive carbohydrate determinants (CCD). This study investigates 105 pollen allergic subjects for the presence of specific IgE to honeybee or wasp venom, pollen, the MUXF3 carbohydrate epitope from bromelain and recombinant Hymenoptera venom components. In addition, in a subgroup of patients (n = 10) a basophil activation test (BAT) using bee and wasp venom was performed. Specific IgE to Hymenoptera venom was detected in 45.7% of the pollen allergic subjects and in 26.7% of the non-atopic controls, both without a history of systemic allergic reactions to Hymenoptera stings. The high sensitization rate in atopic patients could partially be explained by cross-sensitization between pollen and Hymenoptera venom due to specific IgE to CCDs. In our study population, only 20% showed a sensitization to CCDs. Primary sensitization due to sting exposure, high total IgE values or unspecific binding and detection of low affinity antibodies in the test procedure could be reasons. Thus, determination of specific IgE to Hymenoptera venom in patients without a history of systemic allergic reactions as screening test is not recommended.

## Introduction

About 9.8% of the Swiss population shows specific IgE (sIgE) to honeybee or wasp venom without reporting systemic symptoms after Hymenoptera stings [1]. In European epidemiological studies the sensitization rate to honeybee or wasp venom in the general population varies between 27.1–44.1% [2, 3]. Despite this high sensitization rate only 3.5% develop a systemic reaction after a Hymenoptera sting in Switzerland [1], in western countries the rate lies between 0.3–7.5% [4].

The high sensitization rate without evidence of a systemic reaction after Hymenoptera stings is partly explained by the presence of antibodies to cross-reacting carbohydrate determinants (CCD) [5–7]. CCDs are epitopes on glycoproteins that are nearly ubiquitously present in plants, pollen as well as in Hymenoptera venoms [7, 8]. They are thought to be responsible for cross-reactivity between potential allergens with negative impact on the correct diagnosis of allergies. This mechanism is primarily involved in persons with double sensitization to bee and wasp venom [9] and/or with polysensitization to pollen allergens [10–12]. Although atopy is not a risk factor for Hymenoptera venom allergy, the occurrence of high total IgE (t-IgE) or sIgE to pollen allergens is associated with an increased rate of sensitization to bee or wasp venom [13]. An increased sensitization rate is also present in subjects with frequent Hymenopteran stings [14] or regular alcohol consumption [15].

The aim of this study was to investigate the frequency of sensitization to Hymenoptera venom in patients with pollen allergy and to analyse whether the sensitization is due to CCDs.

## Methods

Patients were recruited from the allergy units of the Inselspital, Bern University Hospital and Spital Netz Bern Ziegler, and from two allergy outpatient practices, if they met the following criteria: a history of seasonal rhinoconjunctivitis, verified by skin prick tests. Patients with a systemic allergic reaction to a Hymenoptera sting in the past were excluded. In addition 30 subjects without sensitization to aeroallergens and no history of a Hymenoptera venom allergy served as healthy controls.

Skin prick tests were done with commercially available pollen extracts of birch, ash, grass and mugwort (ALK-Abello, Hørsholm, Denmark); a wheal diameter of ≥ 3mm as recommended in the European standards for skin prick testing [16] was considered positive. Serum was collected to analyse sIgE to recombinant major allergens of pollen (rBet v 1, rOle e 1, rPhl p 1/5, rArt v 1), specified according to the patient’s seasonal symptoms.

Total and sIgE to whole honeybee and wasp venom were measured in all study patients and healthy controls. The MUXF3 carbohydrate epitope from bromelain and the recombinant major allergens of Hymenoptera venom (rApi m 1, rVes v 5, rVes v 1) were analysed in all study participants with pollen allergy. Measurements were performed using UniCAP 100 according to the manufacturer’s instructions (Thermo Fisher Scientific Inc., Waltham, Massachusetts, USA). Values of >0.35 kU/l were considered positive. We have used only MUXF3 as CCD marker in this study to avoid interference with other bromelain epitopes. A disadvantage is that the sIgE to bromelain seems to be slightly more sensitive [17].

In a randomly selected group of honeybee or honeybee and wasp venom-sensitized patients (n = 10), a basophil activation test (BAT) with honeybee and wasp venom (Bühlmann Laboratories AG, Schönenbuch, CH) was performed. Basophils were characterized according to their granularity and CCR3 expression on a FACSCanto flow cytometer (Becton Dickinson AG, San Diego, USA). CD63 served as an activation marker and was expressed as the percentage of activated basophils. Anti-IgE (100 ng/mL; Beckman Coulter, Marseille, France), C5a (Sigma-Aldrich, St. Louis, USA) and fMLP (Sigma-Aldrich, St. Louis, USA) served as positive control.

The study was approved by the local ethics committee (Kantonale Ethikkommission Bern), all participating controls and patients signed informed consent. The study was funded by the Ulrich Mueller-Gierok Allergy foundation Bern.

Analysis were performed using Graphpad Prism 8 (GraphPad Software, Inc, La Jolla, Calif). All results are summarized with descriptive statistics. Proportions are expressed in percentage.

## Results

Between 2012 and 2014, 105 patients were included in the study (59 female [56.2%]; mean age 32 years [range: 16-72y]). 52 (49.5%) had a seasonal allergic asthma, and 39 (39%) suffered from an oral allergy syndrome to food. Three (2.8%) patients described large local reactions after Hymenoptera stings, whereas no systemic allergic reaction was reported (Table 1).

**Table 1:** Characteristics of study subjects and controls.

Parameters	Patients with pollen allergy	controls
number of subjects (n)	105	30
mean age (range)	31.7 years (16–72)	45.8 years (19–77)
gender female (%)	46 (43.8%)	17 (56.6%)
seasonal rhinoconjunctivitis	105 (100%)	0
seasonal asthma	52 (49.5%)	0
oral allergy syndrome	39 (39%)	0
sensitization to pollen		
birch	79 (75.9%)	0
ash	70 (67.3%)	0
grass	93 (90.3%)	0
mugwort	36 (34.6%)	0
history of insect venom allergy	0	0
large local reactions	3	0

**Fig 1:**
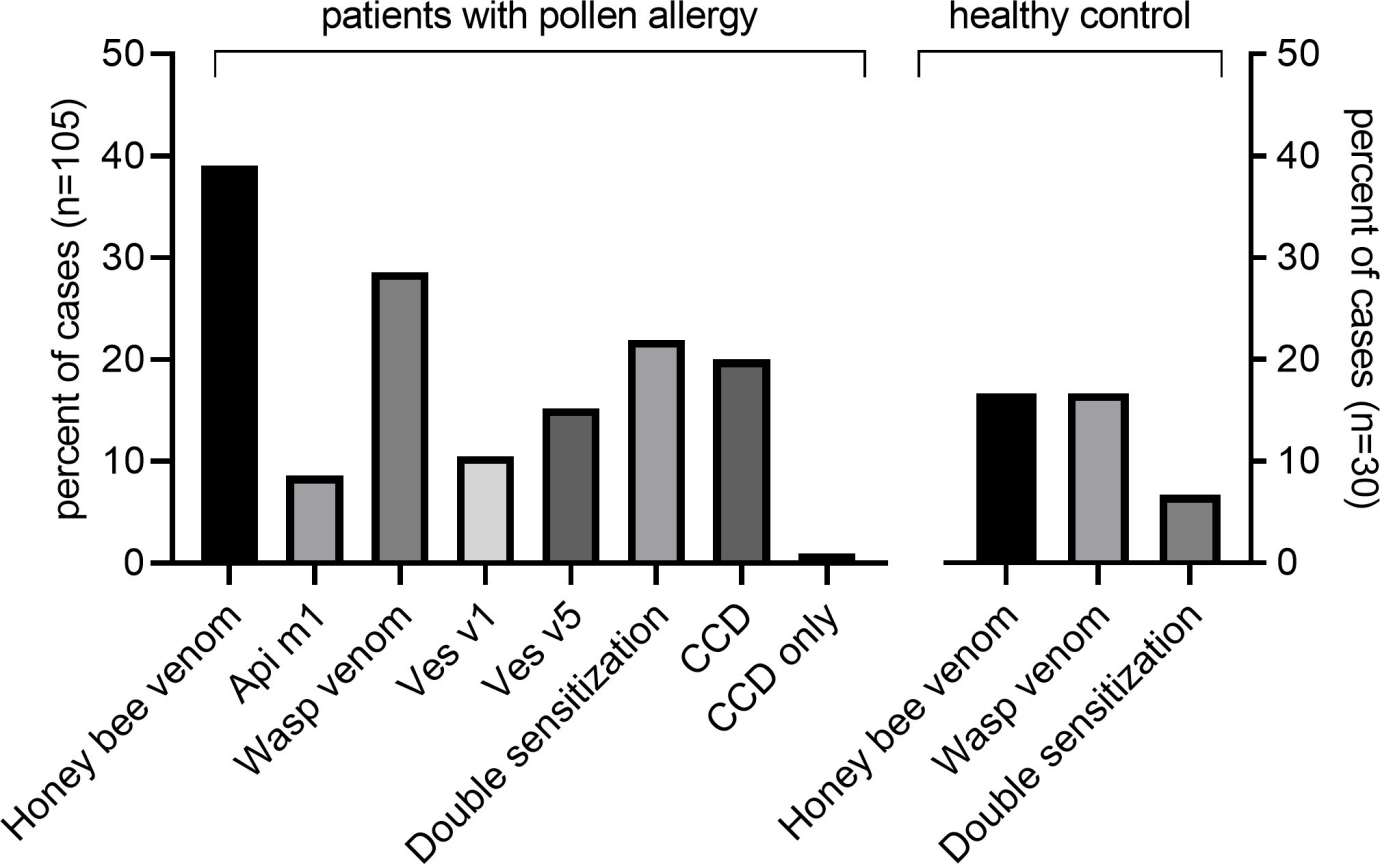
Sensitization rate of study subjects and controls. Sensitization rate of study subjects with pollen allergy (n = 105) and healthy controls without sensitization to pollen allergens (n = 30), analysed by UniCAP 100 (Thermo Fisher Scientific Inc.). Values of >0.35 kU/l were considered positive.

**Fig 2:**
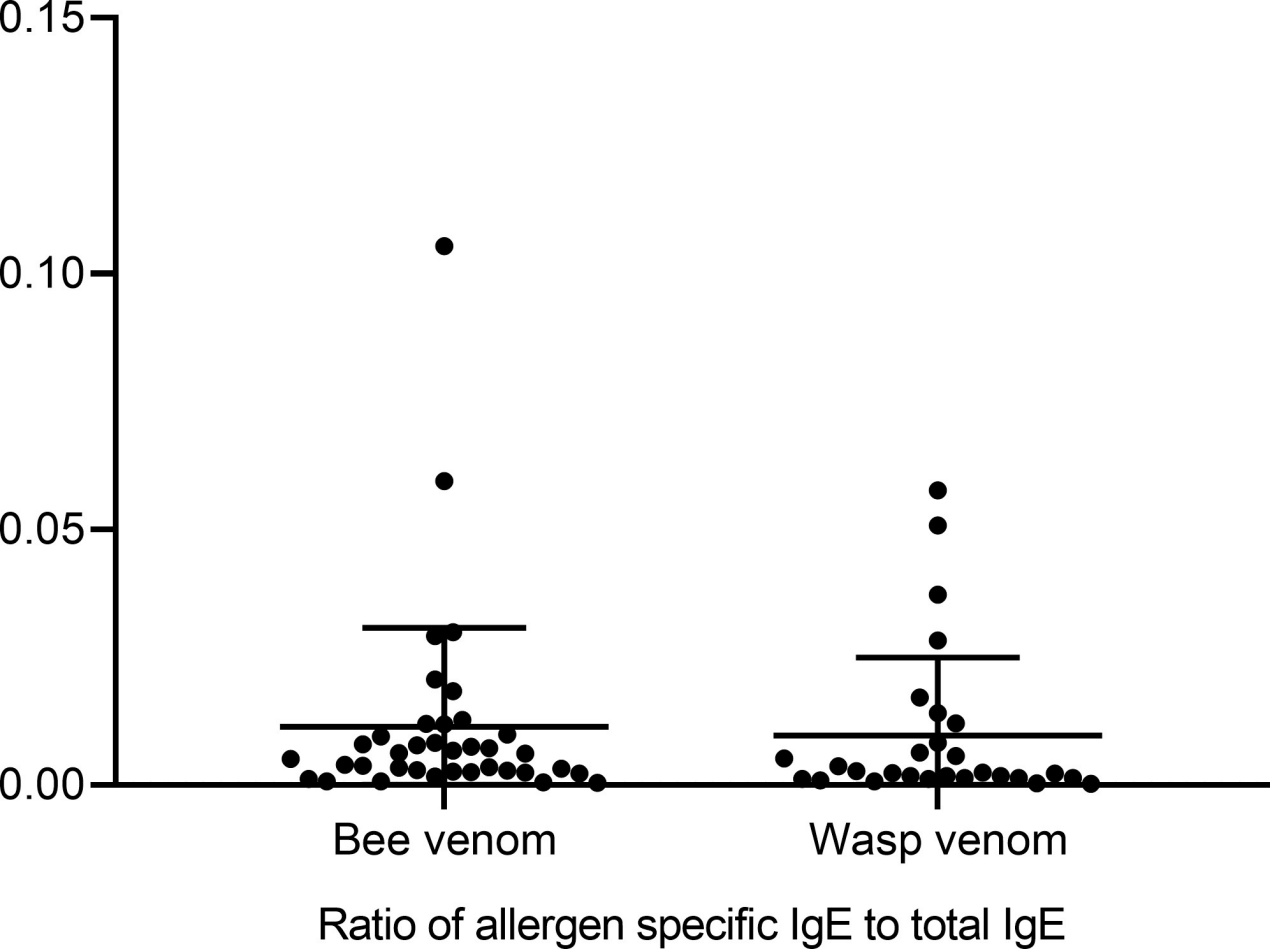
Hymenoptera venom specific IgE/total IgE ratio in subjects with pollen allergy.

48/105 (45.7%) had sIgE to Hymenoptera venoms: 41 (39.0%) of the patients showed sIgE to honeybee venom, 30 (28.6%) to wasp venom and 23 (21.9%) to both venoms. Moreover, 21 (20.0%) of the subjects revealed sIgE to MUXF3, 9 (8.6%) to rApi m 1, 16 (15.2%) to rVes v 5, and 11 (10.5%) to rVes v 1 (Fig 1). Three patients revealed sIgE to rVes v 5 and two to rVes v 1 without evidence of sIgE to wasp venom extract. In contrast, all rApi m 1 positive patients were sensitized to bee venom extract. Overall, a wide variety of sensitization patterns to venom allergens was detected. The majority of sIgE values were in CAP class 2 (0.7–3.4 kU/l) (S1 Fig). Since sIgE values are also related to t-IgE we examined the corresponding ratio (sIgE/t-IgE ratio): With one exception the ratio was below 0.1 (Fig 2).

Focussing on the MUXF3 positive patients, 17 (80.9%) had positive sIgE to honeybee and wasp venom, interestingly 13 (61.9%) showed additional sIgE to recombinant Hymenoptera venom allergens.

7 of 27 patients (25.9%) with sensitization to birch, ash, grass and mugwort pollen showed sIgE to MUXF3. All but one subject with sIgE to MUXF3 were sensitized to grass and/or mugwort pollen (S3 Table).

BAT was performed in 4 honeybee and in 6 double sensitized (honeybee and wasp venom sensitized) subjects; 5 of the double sensitized subjects showed sIgE to MUXF3. Only two patients had a positive BAT to honeybee, but not to wasp venom. Whereas one revealed sIgE to honeybee venom only, the other showed sIgE to honeybee, wasp venom and MUXF3. However, both had no sIgE to the recombinant allergen Api m 1. Therefore only 1 out of 5 patients with sIgE to MUXF3 had a positive BAT.

Of the 30 non-atopic controls, 5 (16.7%) revealed sIgE to honeybee and 5 (16.7%) to wasp venom. Only two patients were double-sensitized to both venoms (2, 6.7%). Controls were not tested for sIgE to recombinant Hymenoptera venom or MUXF3.

## Discussion

In our study almost half (45.7%) of the symptomatic pollen allergic patients and 26.7% of the non-atopic controls without history of a systemic reaction after a Hymenoptera sting had sIgE to Hymenoptera venoms. High sensitization rates to Hymenoptera venoms in asymptomatic subjects have been described [2, 3, 9]. IgE antibodies to CCD were found primarily in individuals with double sensitization to bee and wasp venoms as shown in previous studies [9]. Almost three quarters of all persons with sensitization to bee and wasp venom also had sIgE to CCDs (17/23, 73.9%). In these cases, it can be assumed that at least one sensitization to bee or wasp venom might be false positive. Although we suspected that in patients with pollen allergy, antibodies against CCD are mainly responsible for the sensitization to Hymenoptera venoms, this is only partially true and applies mainly for double sensitized subjects [6, 18]. In our study population, only 20% revealed a sensitization to CCDs. As expected, the proportion is higher in patients with pollen allergy than in healthy subjects without atopy. Only 6.7% of the control group were sensitized to both, bee and wasp venoms, which suggests an association to CCDs.

Besides CCDs, the following reasons can be presumed for the high sensitization rate in pollen allergic people:

- 1. High sensitization rate to Hymenoptera venom could be due to previous stings [14] since most of the individuals have been stung at least once in the past. Thus, it could represent a primary but not relevant sensitization to a Hymenoptera venom. This fact relates to the atopic as well as the non-atopic subjects. The finding that 9–15% of our patients had sIgE to recombinant venom allergens, which are free from glycosylated structures, supports this assumption.
- 2. Positive test results due to non-specific bindings in the test procedure [7, 9] or the detection of low-affinity, cross-reacting sIgE using a high amount of Hymenoptera venoms in in-vitro diagnosis [2] can be assumed.
- 3. High t-IgE levels may influence the rate of sensitization to bee and wasp venoms [13, 19]. Indeed, none of the 22 subjects in our study with a t-IgE <50 IU/l showed sIgE to Hymenoptera venoms whereas 24 of 31 (77.4%) with a t-IgE >250 IU/l had sIgE to Hymenoptera venoms.

With regard to BAT, a positive result to bee venom was found in one out of five cases with sensitization to CCDs. Although no sensitization to the bee major allergen Api m 1 was found in this case, other major allergens (e.g. Api m 10) may have been relevant [20]. Nevertheless, the influence of Anti-CCD IgE on the BAT is probably limited, but could still influence the specificity.

Our study has some limitations: The clinical relevance of the documented sensitizations in our study population is uncertain, but none had a systemic reaction to a Hymenoptera sting in the past. Since the sIgE/t-IgE ratio was low in all cases, a low clinical relevance can be assumed. Sting challenges were not planned as this is not approved by Swiss ethical standards. Regarding bee venom, only the major allergen Api m 1 was analyzed. As other major allergens are relevant too, the proportion of persons with sensitization to recombinant allergens may have been underestimated [20]. In our control group CCD antibodies and recombinant allergens were not analysed, therefore, a comprehensive comparison between pollen allergic individuals and controls was not achievable. Since the control group only included patients without atopy, we assumed that CCDs did play a minor role.

## Conclusion

Besides CCDs other reasons such as a primary sensitization, high t-IgE values or methodological factors have to be considered for the high prevalence of sensitization to Hymenoptera venoms in both pollen allergic and non-allergic individuals. Anti-CCD IgE could lead to false positivity in different detection methods (serology, basophil activation test) and limit their specificity. For the everyday clinical use, serological analyses of sIgE to Hymenoptera venom without a history of systemic reaction are not of value to identify patients at risk. In contrary, due to the absent clinic it rather confuses the patient as well as the treating doctor.

## Supporting information

S1 Fig Specific IgE to bee and wasp venom, CCD and recombinant venom components. Specific IgE to bee and wasp venom, CCD and recombinant venom components in study subjects with pollen allergy (n = 105) and healthy controls without sensitization to pollen allergens (n = 30), analysed by UniCAP 100 (Thermo Fisher Scientific Inc.).
(TIF)

S1 Table Dataset of specific IgE to bee and wasp venom, CCD and recombinant venom components in study subjects with pollen allergy (n = 105). (DOCX)

S2 Table Dataset of specific IgE to bee and wasp venom in healthy controls (n = 30). (DOCX)

S3 Table Dataset of pollen sensitization in study subjects with pollen allergy (n = 105). (DOCX)
