# Supplementary figures and images for: Sensitization to Hymenoptera venom in pollen allergic patients: Frequency and involvement of cross-reacting carbohydrate determinants (CCD)
Source: PLoS One. 2020 Sep 8;15(9):e0238740. doi: 10.1371/journal.pone.0238740 (PMC7478646; doi:10.1371/journal.pone.0238740)

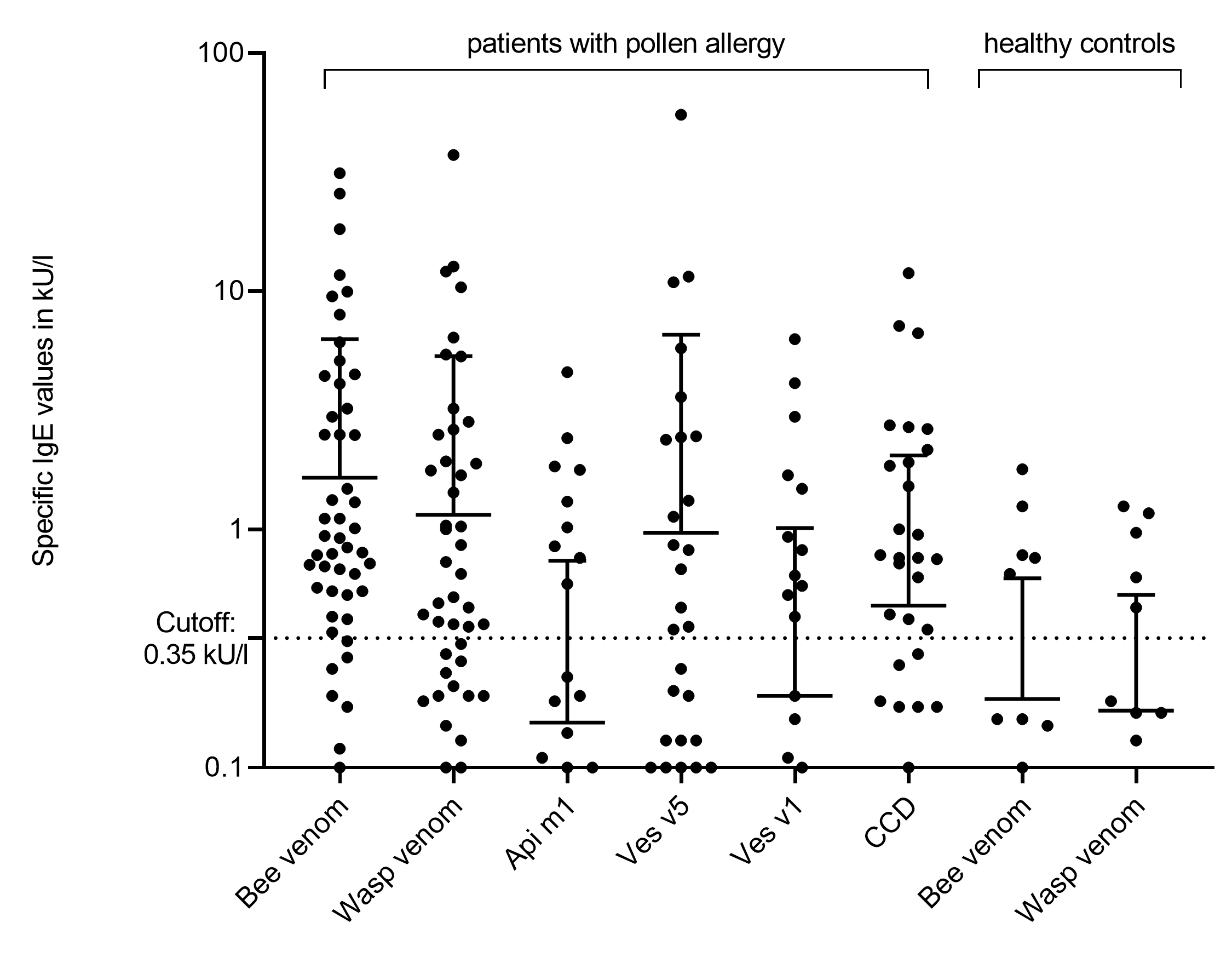

Supplement: S1 Fig — Specific IgE to bee and wasp venom, CCD and recombinant venom components in study subjects with pollen allergy (n = 105) and healthy controls without sensitization to pollen allergens (n = 30), analysed by UniCAP 100 (Thermo Fisher Scientific Inc.). (TIF) [file pone.0238740.s001.tif]
