# Supplementary material for: Sensitization to Hymenoptera venom in pollen allergic patients: Frequency and involvement of cross-reacting carbohydrate determinants (CCD)
Source: PLoS One. 2020 Sep 8;15(9):e0238740. doi: 10.1371/journal.pone.0238740 (PMC7478646; doi:10.1371/journal.pone.0238740)
Supplement: S1 Table — (DOCX) [file pone.0238740.s002.docx]

**S1 table. Dataset of specific IgE to bee and wasp venom, CCD and recombinant venom components in study subjects with pollen allergy (n=105).**

| **Patient** | **Total IgE  kU/l** | **sIgE to bee  venom kU/l** | **sIgE to wasp  venom kU/l** | **sIgE to  Api m 1 kU/l** | **sIgE to  Ves v 5 kU/l** | **sIgE to  Ves v 1 kU/l** | **sIgE to  rMux f 3 kU/l** |
| --- | --- | --- | --- | --- | --- | --- | --- |
| 1 | 53.3 | 0 | 0 | 0 | 0 | 0 | 0 |
| 2 | 79.5 | 0 | 0 | 0 | 0 | 0 | 0 |
| 3 | 420 | 0 | 0 | 0 | 0.82 | 0 | 0 |
| 4 | 163 | 1.33 | 1.03 | 0 | 0.86 | 0 | 0 |
| 5 | 40.3 | 0 | 0 | 0 | 0 | 0 | 0 |
| 6 | 33 | 0.42 | 0 | 0 | 0 | 0.64 | 0 |
| 7 | 76 | 0 | 0 | 0 | 0 | 0 | 0 |
| 8 |  | 0 | 0 | 0 | 0 | 0 | 0 |
| 9 | 323 | 0.72 | 0 | 0 | 0 | 0 | 0 |
| 10 |  | 0 | 0 | 0 | 0 | 0 | 0 |
| 11 | 17 | 1.01 | 0 | 0 | 0 | 0 | 0 |
| 12 |  | 0 | 0 | 0 | 0 | 0 | 0 |
| 13 |  | 0.65 | 0.52 | 0 | 0 | 0 | 0 |
| 14 |  | 1.11 | 0 | 0.59 | 0 | 0 | 1.52 |
| 15 |  | 0.79 | 0 | 0 | 0 | 0 | 0 |
| 16 | 25.2 | 0 | 0 | 0 | 0 | 0 | 0 |
| 17 | 78.5 | 0.78 | 0 | 0 | 0 | 0 | 0 |
| 18 | 111 | 11.7 | 6.39 | 0 | 5.77 | 0 | 6.66 |
| 19 | 360 | 0 | 0.49 | 0 | 0 | 0 | 0 |
| 20 | 1463 | 2.5 | 0.4 | 0 | 0 | 0 | 0 |
| 21 | 37.9 | 0 | 0 | 0 | 0 | 0 | 0 |
| 22 | 11 | 0 | 0 | 0 | 0 | 0 | 0 |
| 23 | 203 | 0 | 0 | 0 | 0 | 0 | 0 |
| 24 | 231 | 0 | 0 | 0 | 0 | 0 | 0 |
| 25 | 377 | 1.11 | 5.32 | 0 | 11.5 | 0 | 0.76 |
| 26 | 25.6 | 0 | 0 | 0 | 0 | 0 | 0 |
| 27 | 59 | 0 | 0 | 0 | 0 | 0 | 0 |
| 28 | 64.2 | 0 | 0 | 0 | 0 | 0 | 0 |
| 29 | 21.7 | 0 | 0 | 0 | 0 | 0 | 0 |
| 30 | 19 | 0 | 0 | 0 | 0 | 0 | 0 |
| 31 | 155 | 0.53 | 0 | 0 | 0 | 0 | 0 |
| 32 | 543 | 0 | 1 | 0 | 2.44 | 0 | 0 |
| 33 | 18.1 | 0 | 0 | 0 | 0 | 0 | 0 |
| 34 |  | 2.5 | 1.43 | 0 | 0 | 0 | 2.16 |
| 35 | 335 | 0.8 | 0 | 0 | 0 | 0 | 0 |
| 36 | 14.3 | 0 | 0 | 0 | 0 | 0 | 0 |
| 37 | 776 | 0.55 | 0.73 | 0 | 0 | 0 | 1.85 |
| 38 | 173 | 0 | 0 | 0 | 0 | 0 | 0 |
| 39 | 36 | 0 | 0 | 0 | 0 | 0 | 0 |
| 40 | 482 | 0 | 0 | 0 | 0 | 0 | 0 |
| 41 | 624 | 4.48 | 0 | 0 | 0 | 0 | 0 |
| 42 | 929 | 0.37 | 1.69 | 0 | 0.39 | 1.48 | 0 |
| 43 | 156 | 3.22 | 1.89 | 0.85 | 0 | 0 | 0.38 |
| 44 | >5000 | 25.7 | 12.1 | 1.31 | 0 | 0 | 7.15 |
| 45 |  | 6.1 | 0 | 0 | 0 | 0 | 0 |
| 46 | 371 | 4.41 | 1.93 | 1.02 | 1.13 | 0.82 | 0.19 |
| 47 | 979 | 2.49 | 1.77 | 0.14 | 0.1 | 0.93 | 0.44 |
| 48 | 241 | 0.92 | 0 | 0.11 | 0 | 0 | 0.75 |
| 49 | 2282 | 7.98 | 3.22 | 0.76 | 0.13 | 0.16 | 2.64 |
| 50 | 334 | 0 | 0 | 0 | 0 | 0 | 0.18 |
| 51 | 318 | 9.51 | 5.43 | 4.58 | 2.46 | 4.11 | 0.95 |
| 52 | 1939 | 1.3 | 0.41 | 0 | 0.1 | 0 | 0.42 |
| 53 | 1911 | 18.2 | 2.62 | 0 | 1.32 | 0.11 | 1.91 |
| 54 | 26 | 0 | 0 | 0 | 0 | 0 | 0 |
| 55 | 177 | 0 | 0 | 0 | 0.68 | 0 | 0 |
| 56 | 76.2 | 0 | 0 | 0 | 0 | 0 | 0 |
| 57 | 100 | 0 | 2.83 | 0 | 2.38 | 0 | 0 |
| 58 | 36.5 | 0 | 0 | 0 | 0 | 0 | 0 |
| 59 | 66.1 | 0 | 0 | 0 | 0 | 0 | 0 |
| 60 | 34.3 | 0 | 0 | 0 | 0 | 0 | 0 |
| 61 | 662 | 0.84 | 0.47 | 0.2 | 0.47 | 0 | 0.76 |
| 62 | 224 | 4.09 | 0 | 2.42 | 0 | 0.43 | 0 |
| 63 | 239 | 0.68 | 0 | 0 | 0 | 0 | 0 |
| 64 | 74.1 | 0 | 0 | 0 | 0.13 | 0 | 0 |
| 65 | 58.5 | 0.7 | 0 | 0 | 0 | 0 | 0 |
| 66 | 481 | 2.97 | 0.1 | 0 | 0 | 0 | 0.18 |
| 67 | 69 | 0 | 0 | 0 | 0 | 0 | 0 |
| 68 | 88.4 | 0 | 0 | 0 | 0 | 0 | 0 |
| 69 | 94.1 | 0.57 | 0 | 0.24 | 0 | 0 | 0 |
| 70 | 127 | 0 | 1.04 | 0 | 0.21 | 0.53 | 0 |
| 71 | 114 | 0 | 0.65 | 0 | 0 | 0.58 | 0 |
| 72 | 172 | 0.55 | 0.4 | 0 | 0 | 0 | 0.72 |
| 73 | 736 | 0.34 | 37.3 | 0 | 55.1 | 6.29 | 0.63 |
| 74 | 29.2 | 0.18 | 0.19 | 0 | 0 | 0 | 0 |
| 75 | 367 | 1.48 | 0.44 | 0.19 | 0 | 0 | 0.27 |
| 76 | 63.8 | 0 | 0.2 | 0 | 0 | 0 | 0 |
| 77 | 476 | 0 | 0 | 0 | 0 | 0 | 2.74 |
| 78 | 16.6 | 0 | 0 | 0 | 0 | 0 | 0 |
| 79 | 197 | 0 | 0 | 0 | 0 | 0 | 0 |
| 80 | 51.9 | 0 | 0.28 | 0 | 0.26 | 0 | 0 |
| 81 | 268 | 0 | 0 | 0 | 0 | 0 | 0 |
| 82 | 209 | 0.12 | 0 | 0 | 0 | 0 | 0 |
| 83 | 25.2 | 0.1 | 0 | 0 | 0 | 0 | 0 |
| 84 | 20.8 | 0 | 0 | 0 | 0 | 0 | 0 |
| 85 | 258 | 0 | 0.25 | 0 | 0.13 | 0 | 0 |
| 86 | 298 | 0.26 | 0.13 | 0 | 0 | 0 | 0.18 |
| 87 | 36.5 | 0 | 0 | 0 | 0 | 0 | 0 |
| 88 | 275 | 0.71 | 0.39 | 0 | 0 | 0 | 0.78 |
| 89 | 27.6 | 0 | 0.33 | 0 | 0 | 0 | 0 |
| 90 | 27.9 | 0 | 0.1 | 0 | 0.1 | 0 | 0 |
| 91 | 241 | 0 | 0.22 | 0 | 0 | 0 | 0 |
| 92 | 315 | 0 | 0.86 | 0 | 0 | 0 | 0 |
| 93 | 341 | 9.94 | 12.7 | 1.78 | 10.9 | 1.69 | 1 |
| 94 | 70.9 | 0 | 0 | 0 | 0 | 0 | 0 |
| 95 | 2063 | 0.29 | 0 | 0 | 0.1 | 0 | 0.1 |
| 96 | 4698 | 31.3 | 10.4 | 1.84 | 3.6 | 2.97 | 11.9 |
| 97 | 152 | 0 | 0 | 0 | 0 | 0 | 0 |
| 98 | 682 | 5.1 | 2.5 | 0.1 | 0 | 0 | 2.69 |
| 99 |  | 0 | 0 | 0 | 0 | 0 | 0 |
| 100 | 140 | 0 | 0 | 0 | 0 | 0 | 0 |
| 101 | >5000 | 0.2 | 0.2 | 0.1 | 0.2 | 0.2 | 0.3 |
| 102 | 120 | 0.94 | 0.3 | 0 | 0.1 | 0.1 | 0 |
| 103 | 26.9 | 0 | 0 | 0 | 0 | 0 | 0 |
| 104 | 53.8 | 0.43 | 0.2 | 0 | 0.38 | 0 | 0 |
| 105 | 33.6 | 0 | 0.15 | 0 | 0 | 0 | 0 |
