# Supplementary material for: Sensitization to Hymenoptera venom in pollen allergic patients: Frequency and involvement of cross-reacting carbohydrate determinants (CCD)
Source: PLoS One. 2020 Sep 8;15(9):e0238740. doi: 10.1371/journal.pone.0238740 (PMC7478646; doi:10.1371/journal.pone.0238740)
Supplement: S2 Table — (DOCX) [file pone.0238740.s003.docx]

**S2 table. Dataset of specific IgE to bee and wasp venom in healthy controls (n=30).**

| **Control** | **sIgE to bee venom kU/l** | **sIgE to wasp venom kU/l** |
| --- | --- | --- |
| 1 | 1.79 | 0.19 |
| 2 | 0 | 0.63 |
| 3 | 0 | 0 |
| 4 | 0 | 0 |
| 5 | 0 | 0 |
| 6 | 0.78 | 0.13 |
| 7 | 0.1 | 0.17 |
| 8 | 0.16 | 0 |
| 9 | 0 | 0 |
| 10 | 0 | 0 |
| 11 | 0 | 0 |
| 12 | 0 | 0 |
| 13 | 0.76 | 0.47 |
| 14 | 0.16 | 1.25 |
| 15 | 0 | 0 |
| 16 | 0 | 0.17 |
| 17 | 0 | 0 |
| 18 | 0 | 0 |
| 19 | 0.15 | 0.97 |
| 20 | 0 | 0 |
| 21 | 0 | 0 |
| 22 | 0 | 0 |
| 23 | 0 | 0 |
| 24 | 0 | 0 |
| 25 | 0 | 0 |
| 26 | 0 | 0 |
| 27 | 1.25 | 1.17 |
| 28 | 0.02 | 0 |
| 29 | 0.65 | 0.04 |
| 30 | 0.01 | 0.01 |
