# Supplementary material for: Sensitization to Hymenoptera venom in pollen allergic patients: Frequency and involvement of cross-reacting carbohydrate determinants (CCD)
Source: PLoS One. 2020 Sep 8;15(9):e0238740. doi: 10.1371/journal.pone.0238740 (PMC7478646; doi:10.1371/journal.pone.0238740)
Supplement: S3 Table — (DOCX) [file pone.0238740.s004.docx]

**S3 table. Dataset of pollen sensitization in study subjects with pollen allergy (n=105).**

^¶^ Study subjects with specific IgE to MUXF3 (cutoff >0.35 kU/l)

| **Patient** | **Birch pollen** | **Ash tree pollen** | **Grass pollen** | **Mugwort pollen** |
| --- | --- | --- | --- | --- |
| 1 | yes | yes | yes | yes |
| 2 | yes | yes | yes | yes |
| 3 | yes | yes | yes | yes |
| 4 | yes | yes | yes | - |
| 5 | yes | - | yes | - |
| 6 | - | - | yes | - |
| 7 | yes | yes | yes | - |
| 8 | yes | yes | yes | - |
| 9 | yes | yes | yes | yes |
| 10 | yes | yes | yes | - |
| 11 | yes | yes | yes | yes |
| 12 | yes | - | yes | - |
| 13 | yes | yes | yes | - |
| 14^¶^ | yes | yes | yes | - |
| 15 | - | yes | yes | yes |
| 16 | - | yes | yes | - |
| 17 | - | - | yes | - |
| 18^¶^ | - | - | yes | yes |
| 19 | yes | - | yes | - |
| 20 | yes | yes | yes | yes |
| 21 | yes | yes | yes | - |
| 22 | yes | - | - | - |
| 23 | yes | - | - | - |
| 24 | yes | yes | yes | yes |
| 25^¶^ | yes | - | - | yes |
| 26 | yes | - | yes | - |
| 27 | yes | yes | - | - |
| 28 | yes | yes | yes | yes |
| 29 | yes | - | - | - |
| 30 | yes | yes | yes | yes |
| 31 | - | yes | yes | yes |
| 32 | yes | yes | yes | yes |
| 33 | yes | - | - | - |
| 34^¶^ | yes | yes | yes | yes |
| 35 | yes | yes | yes | - |
| 36 | yes | - | - | - |
| 37^¶^ | yes | - | yes | - |
| 38 | yes | yes | yes | - |
| 39 | yes | - | yes | - |
| 40 | yes | yes | yes | yes |
| 41 | yes | yes | yes | - |
| 42 | yes | yes | yes | yes |
| 43^¶^ | - | yes | yes | - |
| 44^¶^ | yes | yes | yes | - |
| 45 | yes | yes | - | - |
| 46 | yes | yes | yes | yes |
| 47^¶^ | yes | - | yes | - |
| 48^¶^ | yes | yes | yes | yes |
| 49^¶^ | - | - | yes | - |
| 50 | yes | yes | yes | - |
| 51^¶^ | yes | yes | yes | yes |
| 52^¶^ | yes | yes | yes | - |
| 53^¶^ | yes | yes | yes | yes |
| 54 | yes | yes | yes | - |
| 55 | yes | - | yes | yes |
| 56 | yes | yes | yes | - |
| 57 | - | yes | yes | - |
| 58 | yes | yes | yes | yes |
| 59 | - | yes | yes | - |
| 60 | yes | yes | yes | - |
| 61^¶^ | yes | yes | yes | yes |
| 62 | - | yes | yes | - |
| 63 | yes | yes | yes | - |
| 64 | - | - | - | yes |
| 65 | - | - | yes | yes |
| 66 | - | - | yes | - |
| 67 | - | yes | yes | - |
| 68 | yes | yes | yes | - |
| 69 | yes | yes | yes | - |
| 70 | yes | yes | yes | - |
| 71 | yes | - | yes | - |
| 72^¶^ | yes | yes | yes | yes |
| 73^¶^ | yes | yes | yes | - |
| 74 | - | - | yes | - |
| 75 | yes | yes | yes | yes |
| 76 | - | yes | yes | - |
| 77^¶^ | yes | yes | yes | yes |
| 78 | yes | - | yes | - |
| 79 | yes | yes | yes | yes |
| 80 | - | - | yes | - |
| 81 | yes | yes | yes | - |
| 82 | - | - | yes | - |
| 83 | - | yes | yes | - |
| 84 | yes | yes | yes | - |
| 85 | yes | - | yes | - |
| 86 | yes | yes | yes | yes |
| 87 | - | - | yes | - |
| 88^¶^ | yes | - | yes | yes |
| 89 | yes | yes | yes | - |
| 90 | yes | yes | yes | yes |
| 91 | yes | yes | yes | yes |
| 92 | yes | yes | yes | - |
| 93^¶^ | yes | yes | - | - |
| 94 | yes | yes | yes | yes |
| 95 | yes | yes | yes | yes |
| 96^¶^ | - | yes | yes | - |
| 97 | yes | - | - | - |
| 98^¶^ | - | - | yes | - |
| 99 | yes | yes | yes | - |
| 100 | yes | - | yes | - |
| 101 | - | - | yes | - |
| 102 | - | - | yes | - |
| 103 | - | - | yes | - |
| 104 | yes | yes | yes | - |
| 105 | yes | yes | - | - |
